# Microwave bone fracture diagnosis using deep neural network

**DOI:** 10.1038/s41598-023-44131-5

**Published:** 2023-10-07

**Authors:** Sina Beyraghi, Fardin Ghorbani, Javad Shabanpour, Mir Emad Lajevardi, Vahid Nayyeri, Pai-Yen Chen, Omar M. Ramahi

**Affiliations:** 1 Department of Information and Communications Technologies, Pompeu Fabra University, Barcelona, Spain; 2 School of Electrical Engineering, Iran University of Science and Technology, Tehran, 1684613114 Iran; 3 Department of Electronics and Nanoengineering, School of Electrical Engineering, Aalto University, 02150 Espoo, Finland; 4 Department of Electrical Engineering, Faculty of Electrical and Electronics, South Tehran Branch, Islamic Azad University, Tehran, 113654435 Iran; 5 School of Advanced Technologies, Iran University of Science and Technology, Tehran, 1684613114 Iran; 6 Department of Electrical and Computer Engineering, University of Illinois, Chicago, IL 60607 USA; 7 Department of Electrical and Computer Engineering, University of Waterloo, Waterloo, N2L3G1 Canada

**Keywords:** Biomedical engineering, Diagnosis

## Abstract

This paper studies the feasibility of a deep neural network (DNN) approach for bone fracture diagnosis based on the non-invasive propagation of radio frequency waves. In contrast to previous “semi-automated” techniques, where X-ray images were used as the network input, in this work, we use S-parameters profiles for DNN training to avoid labeling and data collection problems. Our designed network can simultaneously classify different complex fracture types (normal, transverse, oblique, and comminuted) and estimate the length of the cracks. The proposed system can be used as a portable device in ambulances, retirement houses, and low-income settings for fast preliminary diagnosis in emergency locations when expert radiologists are not available. Using accurate modeling of the human body as well as changing tissue diameters to emulate various anatomical regions, we have created our datasets. Our numerical results show that our design DNN is successfully trained without overfitting. Finally, for the validation of the numerical results, different sets of experiments have been done on the sheep femur bones covered by the liquid phantom. Experimental results demonstrate that fracture types can be correctly classified without using potentially harmful and ionizing X-rays.

## Introduction

Bone fractures can be defined as partial or complete breaking in the continuity of the bones. Fractures in the tibia, which is the longest bone of the human body, are the most common, especially among children, athletes, and aged people, which are challenging to diagnose. Therefore, a fast and accurate diagnosis of bone fracture can boost the speed of the healing process^1^.

X-ray, computed tomography (CT), and magnetic resonance imaging (MRI) are the most used imaging methods for different illnesses, particularly fracture detection^2–4^. X-rays are the most common and widely available diagnostic imaging technique in which the part of the body being pictured is exposed by X-rays. Although X-rays images are not very clear, they are sufficient for diagnosing fractured bones^5^. To produce a more detailed, cross-sectional image of the body, CT using X-rays is used; however, at the cost of exposure to high-level ionizing radiation, which can potentially endanger the patient’s health if repeated frequently. Unlike X-rays and CT scans, MRI works without X-ray radiation. An MRI scanner generates images of the organs within the body using a strong static magnetic field and pulsed radio waves. In addition to being very expensive and time-consuming, MRIs can be detrimental to the operation of certain medical devices and implants. There is also evidence that MRI has biological effects requiring safety consideration^6,7^.

Microwave imaging and sensing is an alternative medical diagnosis method based on nonionizing electromagnetic signals ranging in frequency from hundreds of megahertz to tens of gigahertz. It has the advantages of low health risk, low-cost implementation, low operational cost, and ease of use^8^. This technique relies on the difference between different tissues’ dielectric properties (permittivity and conductivity), which scatter or interact with electromagnetic waves differently. Several studies investigated the feasibility of using microwaves in various medical applications such as respiration and heartbeat detection, brain imaging, breast cancer detection, heart imaging, bone imaging^8–10^, and, very recently, bone fracture diagnosis^11–15^. However, some challenges prevented these methods from becoming commercially or clinically viable. These challenges include effective coupling of microwave signals to the body, minimal difference between dielectric properties of different tissues, operating frequency (trade-off between the penetration depth and the resolution), and electromagnetic interference and noise^8,9^.

Although CTs and MRIs result in high-resolution images, interpretation of the images needs the opinion of expert radiologists. In fact, images can be misinterpreted^16,17^, and sometimes radiologists use several images to increase the interpretation accuracy^18,19^. Software-defined diagnosis is a potential solution to address these challenges. Several studies have applied artificial intelligence (AI) on X-rays, CT, and MRI images for a fast, inexpensive, intelligent, and accurate medical diagnosis^20–22^, including bone fractures diagnosis^23–25^. In microwave-based medical sensing and imaging, where the resolution, information, and accuracy are lower, the need for AI for accurate diagnosis is higher, so it has been applied for different biological diagnoses^26,27^. However, all the previous works on microwave bone fracture diagnosis^11–15^ are based on theoretical methods and manual classification methods, not using AI.

**Figure 1:**
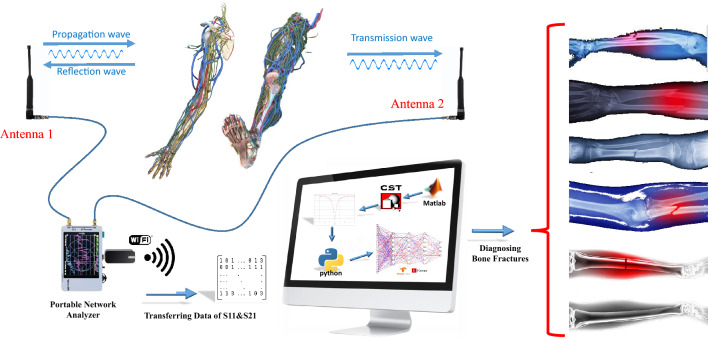
Sketch representation of the proposed Fully-automated bone fracture system using the ISM frequency band. By placing fractured bones in the far-field distance of two monopole antennas, the reflection parameters of the bone can be transferred to the designed DNN. Based on the previously trained data, DNN will decide the fracture types as well as the length of the crack.

This work combines microwave sensing and AI for accurate bone fracture diagnosis. This is accomplished by placing a bone to be tested between two monopole antennas operating in the industrial, scientific, and medical (ISM) frequency band (2.45–2.5 GHz)^28,29^ and investigating the scattered signal from the bone. The type and severity of fracture have a unique response to electromagnetic scattering. Then, a deep learning network learns the exact relation between the scattering parameters and their corresponding defined features. Various types of fractures (normal, transverse, oblique, and comminuted) with different dimensions in different parts of the body (such as the leg, arm, and thigh) are modeled and used to produce a comprehensive dataset. To validate the concept, an experimental phantom-based test setup is established where the sheep femur bone is used for ex-vivo measurements. Additionally, the proposed method is applied to diagnose different types of fractures in the sheep bone.

## Methodologies

### System description

The proposed fully-automated fracture monitoring system comprises two identical commercial dipole antennas (2.4–2.5 GHz) connected to a portable VNA. The power transferred from monopole 1 to monopole 2 can be characterized by the scattering parameters, ${\mathrm{S_{11}}}$ and ${\mathrm{S_{21}}}$, measured by the VNA. Figure 1 shows a sketch representing the concept of using incident and reflected waves to generate datasets using a vector network analyzer (VNA). In the real world, the probability of having identical fractures is extremely low^30^. This reality is expressed by postulating that each type of fracture and different crack dimensions have a unique scattering response.

**Figure 2:**
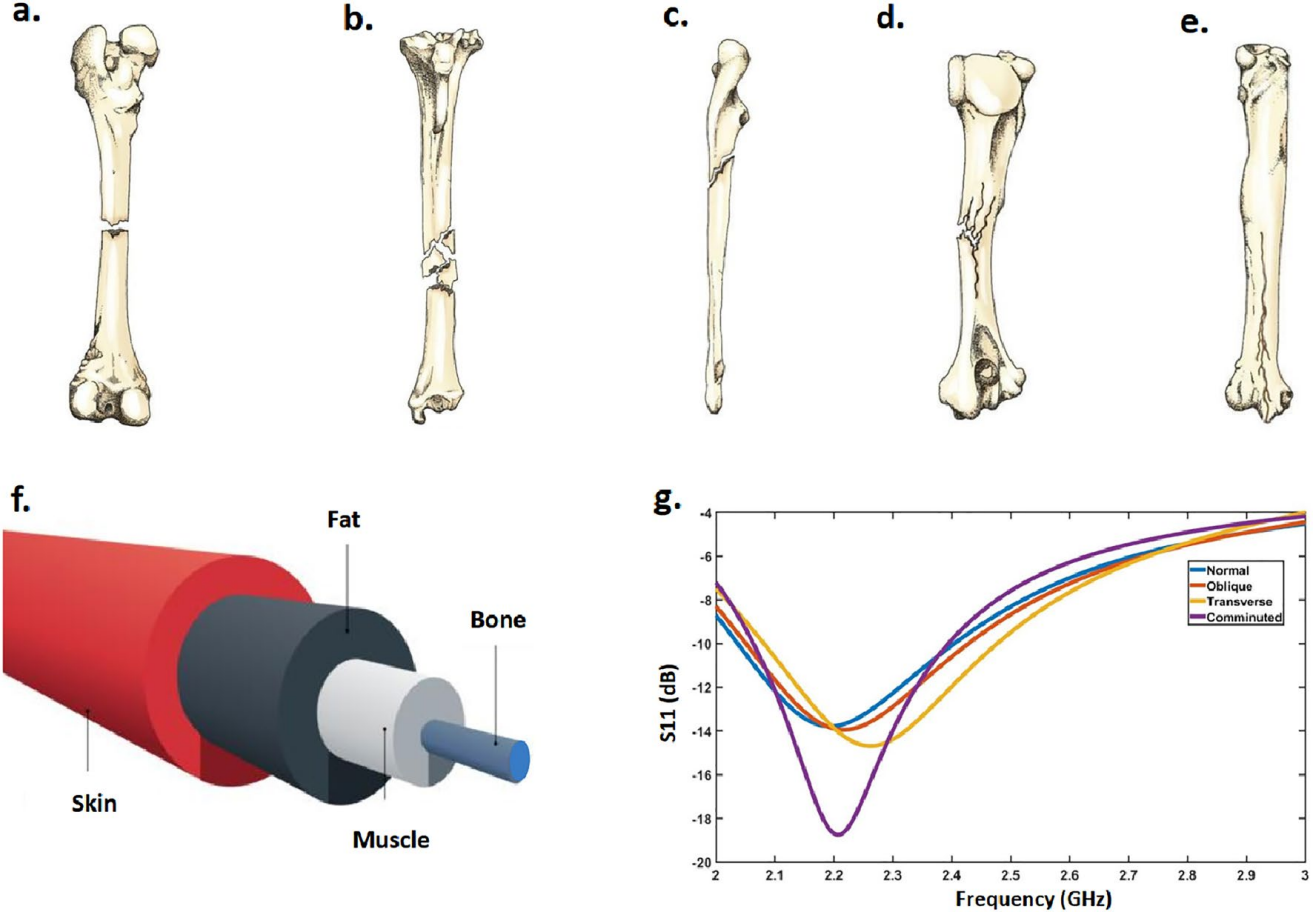
Major fracture types: (**a**) Transverse, (**b**) Comminuted, (**c**) Oblique, (**d**) Greenstick, and (**e**) Fissured fractures. In this paper, we focus on the first three fractures. (**f**) The simulation model of the human arm consists of different cylinders with different diameters. (**g**) The simulation ${\mathrm{S_{11}}}$ results for different types of fractures with the same tissue diameters.

Therefore, by means of monitoring the scattering parameters, our designed deep neural network (DNN) can successfully learn the relation between the S-parameters and their corresponding fractures. The reason originates from the fact that any crack in a bone can be modeled as an air gap. Thus, the S-parameters can be expressed as a function of the effective dielectric constant of the bone and the air gap, which is unique for any specific fracture. Moreover, to broaden the applicability of our method, we also investigate the fractures in different parts of the body (arm, hand, leg, thigh). The EM model of the human body at the ISM frequency band was derived from a previous work^31^.

### Numerical simulation

In order to transition to a fully automated fracture diagnosis and to eliminate the need for labeling data collection from hospitals, numerical simulations are performed on highly accurate models of the human body. The EM simulator CST Microwave Studio® is employed to carry out all the numerical simulations in this study. Figure 2a–e shows the major fracture types. In the present work, we consider three types of fractures, transverse, comminuted, and Oblique, which are depicted in Fig. 2a–c. For numerical simulations, the human arm model is depicted in Fig. 2f, which consists of four layers. Each of the constitutive layers of the arm has its own electromagnetic properties at the ISM band (See Table 2 of^31^). The two antennas are positioned 16 cm away from the fractured bone. The two antennas resonate at 2.45 GHz. By placing the healthy bone model (without fracture), the resonance frequency shifts. Figure 2g illustrates the ${\mathrm{S_{11}}}$ curves for different types of fractures while keeping the diameter of the tissue and the length of the cracks constant.

The data preparation process was conducted by generating a total of 2414 different simulations by considering different fracture types and tissue dimensions. In the generated data, we accounted for four different crack lengths: 0 mm (no fracture), 6 mm, 12 mm, and 18 mm. The bone thicknesses ranged from 5 mm to 25 mm, increasing in 5 mm increments. Additionally, the skin samples had thicknesses of 0.75 mm, 1.5 mm, and 2 mm. For the fat samples, we utilized thicknesses of 3 mm, 12 mm, and 22 mm. Lastly, the muscle thicknesses varied at 30 mm, 50 mm, and 70 mm. Each class, except for the comminuted mode, consisted of 540 data points. To account for the complexity of the comminuted mode, additional simulations were performed by introducing various bone pieces at random positions within the fracture, resulting in a total of 794 data points for this class. One of the major drawbacks of the previous works (data gathering from the X-ray images) is that the collected images would not cover all possible fractures. Simulation-based data collection, on the other hand, gives the flexibility to define any existing fracture or crack’s length using an accurate model of the human body.

### Deep neural network

Deep learning, which is a branch of machine learning that emulates the various activities of a human brain, can automatically learn the association between input and target data from past experiences^32–35^. With neural network architectures, basic principles can be analyzed based on previously provided data, and then, for different inputs, reasonable conclusions can be drawn^36–40^.

The output of a network by the activation function $\phi (u)$ and bias value *b* can be defined as the following, where each input neuron (${X_i}$) has its own weight denoted by ${W_i}$.

1 $$\begin{aligned} Y = \phi \left( {\sum \limits _{i = 1}^n {{W_i}{X_i} + {b_i}} } \right) \ \end{aligned}$$

**Figure 3:**
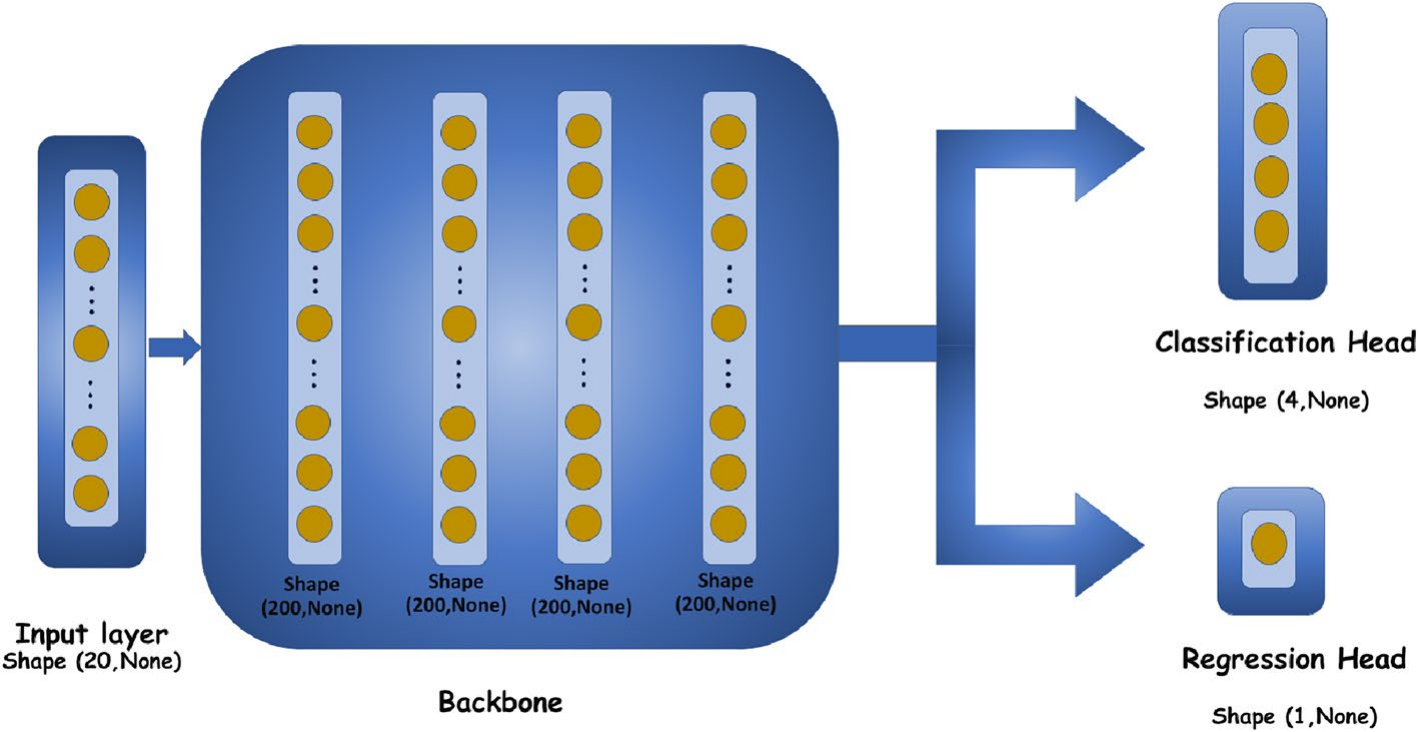
Detailed information on our designed network architecture with two classification and regression outputs.

We have defined the four types of fractures and different tissue dimensions as the varying parameters for producing random datasets. We have generated 2414 sets of random matrices for the data preparation using the “RAND” function of MATLAB. Then, by linking the CST MWS with MATLAB, we calculated the reflection characteristic for each case. The S-parameters are then saved to generate the database. To increase the performance of the network, the DNN was fed with the complete S-parameters information from 2-3 GHz (not only the resonance frequency). We have made an average with a step of 50 MHz in the S-parameters diagram; thus, the input of our proposed DNN is a vector with a dimension of 20 (See Fig. 3).

In the training procedure, 70% of the generated data is considered for training, 15% for testing, and 15% for validation. Also, the datasets are normalized. As depicted in Fig. 3, the output of the training model has two heads. One of them is used as the classification for diagnosis of the types of fractures, while the other one is a regression for estimating the length of the crack if the bone is fractured.

In our proposed DNN, fully connected (dense) layers are used in which each neuron in the input is connected to all the neurons in the former layers. The performance of the machine learning algorithm is enhanced by averaging the input S-parameters at each 50 MHz step. This improvement is achieved by reducing the size of the inputs in the neural network. By reducing the complexity of the model, a smaller number of datasets can be used to achieve the desired accuracy. The main objective of reducing the input size of the deep neural network is to find a balance between model complexity, accuracy, and generalization. As a result of this approach, the model’s performance is improved overall. In the training procedure, we have used the Adam optimizer for training the weighting values. The batch size and the learning rate are equal to 15 and 0.0001, respectively. For regression output, each layer’s weight values were tuned and optimized recurrently to compute the variance between the original and generated data. The ReLU activation function has been employed in all layers of the model, including the head part of the regression. In the head part of the classification, however, the Softmax activation function has been utilized. The training process terminates when the variance reaches a specified benchmark, called a loss function. We have used the Mean Square Error (MSE) as the regression loss function:

2 $$\begin{aligned} MSE = \frac{1}{N}\sum \limits _{i = 1}^N {{{\left( {{{y'}_i} - {y_i}} \right) }^2}} \end{aligned}$$

Above, *N*, ${{{y'}_i}}$ and ${{y_i}}$ denote the number of data points, anticipated value, and the actual value, respectively. We have used categorical cross-entropy as the loss function for the classification output. Our network employs the Relu activation function, except for the last classification layer that uses the Softmax activating function. The formulation of Relu and Softmax activation functions are listed below.

3 $$\begin{aligned} f(z)= & {} \left\{ \begin{array}{l} 0\,\,\,for\,z < 0\\ z\,\,\,for\,z \ge 0 \end{array} \right. \ \end{aligned}$$

4 $$\begin{aligned} \sigma {(\vec {z})_i}= & {} \frac{{{e^{{z_i}}}}}{{\sum \limits _{j = 1}^K {{e^{{z_j}}}} }} \end{aligned}$$

In the Softmax activation function, $\vec {z}$ is an input vector, and *K* denotes the number of classes in the multi-class classifier. In our work, $K=4$ which corresponds to four different types of bone fractures. Tensorflow version 2.2.0 and Keras version 2.3.1 are used to implement the DNN algorithm in Python version 3.9. The number of trainable parameters is 105,625.

**Figure 4:**
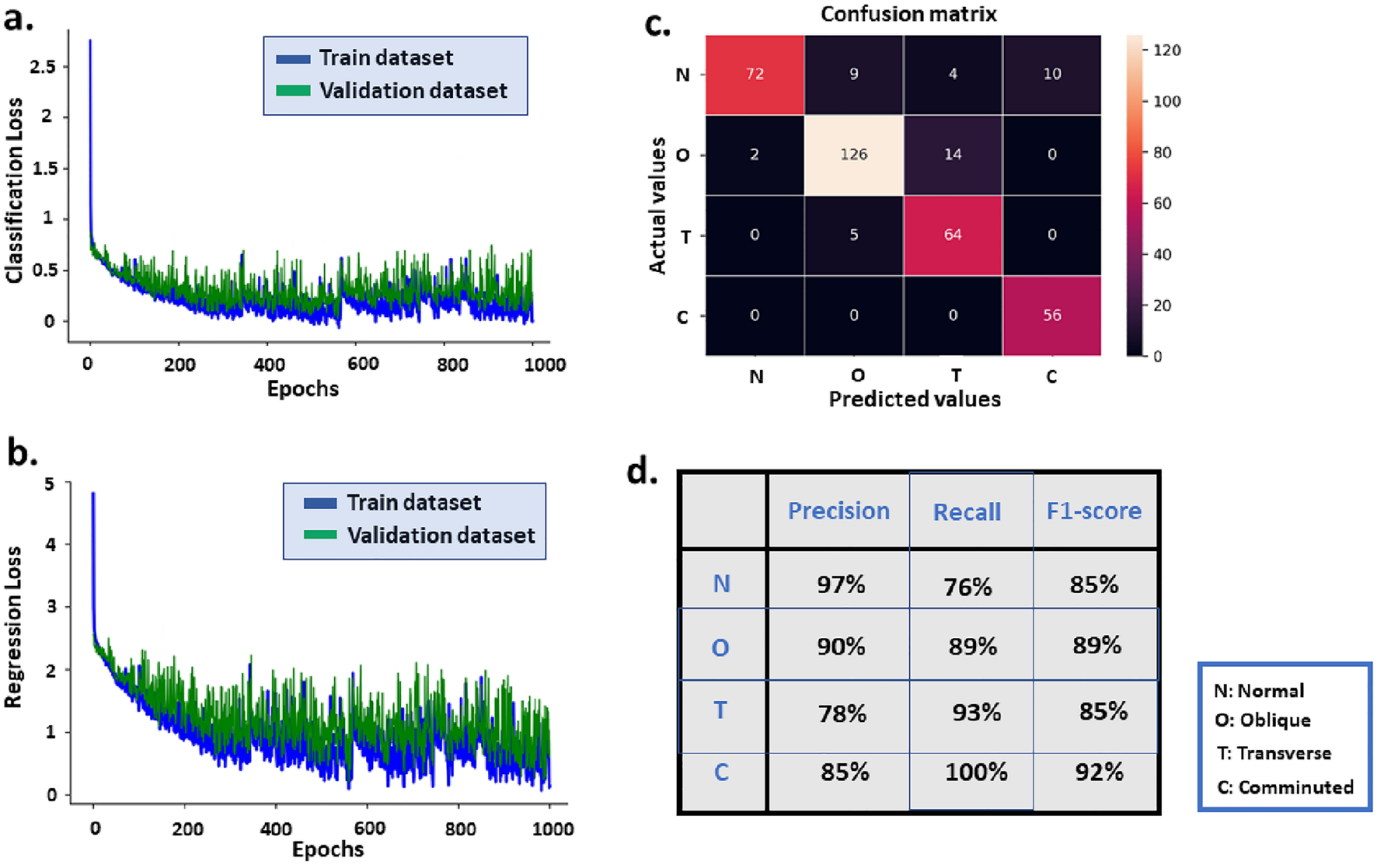
(**a**) Classification, and (**b**) regression loss curves for both training and validation datasets, (**c**) the multi-class confusion matrix, and (**d**) the amount of three different defined metrics for classifier performance evaluation.

## Results and experiments

### Numerical results

The numerical results were obtained using a configuration of Google Colab with a Tesla T4 GPU that has 15 GB of RAM. The training time was 25 minutes, while the evaluation time for classification and regression was 0.11 seconds. Figure 4a shows the curves of the loss function for the classification output (for both training and validation datasets) after 1000 epochs. The correlated dropping of the validation and training loss function after 1000 epochs clearly demonstrates that our design DNN is successfully trained without overfitting. According to Fig. 4b, the same justification can be applied to the loss function for regression output (both training and validation). As we have used two different loss functions for classification and regression output, the loss curves have different values on the y-axis.

Various performance metrics have been adopted to assess the classification networks for fracture diagnoses, such as recall, F-measure, and AUC^41–43^. Among them, the confusion matrix is one of the simplest and most insightful metrics for specifying the accuracy and correctness of a classifier on a test set.

Figure 4c depicts the multi-class confusion matrix where the row and column correspond to the actual and predicted values by DNN. The abbreviations of N, O, T, and C correspond to four different types of bones: normal (without fractures), oblique, transverse, and comminuted fractures. In our multi-class classification problem, we have used precision, recall, and F1-score for DNN performance evaluation. After using numerical simulations on the test dataset and some calculations, the values of precision, Recall, and F1-score for different classes are available in Fig. 4d. Furthermore, the mean regression error on the test dataset is 0.91 mm, with a standard deviation of 1.04 mm. According to the loss curves presented for the training and validation datasets as well as the confusion matrix for the test dataset, we can conclude that our DNN produces accurate and reliable fracture classification results.

### Tissue-equivalent liquid phantom

**Figure 5:**
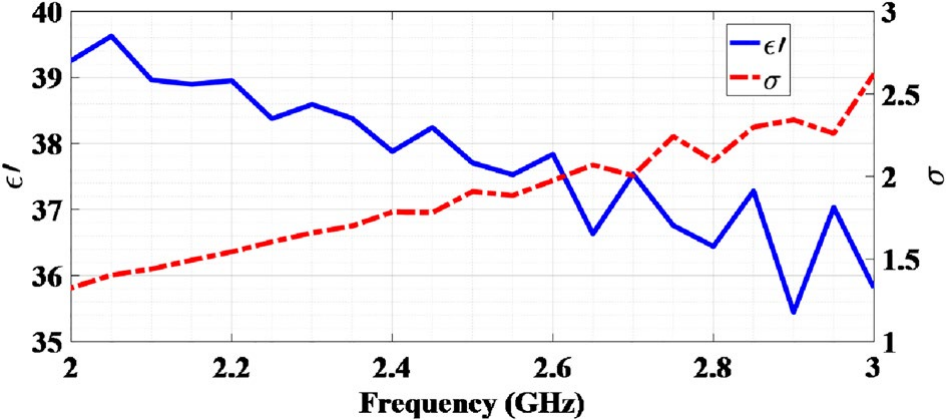
Measured dielectric properties of tissue-equivalent phantom liquid.

Commercial tissue-equivalent liquid phantom is made in accordance with the IEC/IEEE 62209-1528:2020 standard^44^. This liquid comprises deionized water, diethylene glycol butyl ether (DEG), and oxidized mineral oil. As per the standard, the dielectric properties of this phantom, including the real part of the relative permittivity and conductivity at a frequency of 2450 MHz, are $\varepsilon _{r}^{'} = 39.2$ and $\sigma = 1.80$ S/m, respectively. Figure 5 shows the measured dielectric properties of the phantom using a standard coaxial probe and a network vector analyzer. At 2450 MHz, the measured values are $\varepsilon _{r}^{'} = 38.2$ and $\sigma = 1.78$ S/m, showing a high level of agreement with the standard values (with a slight variation of 2.4% and 0.99%).

**Figure 6:**
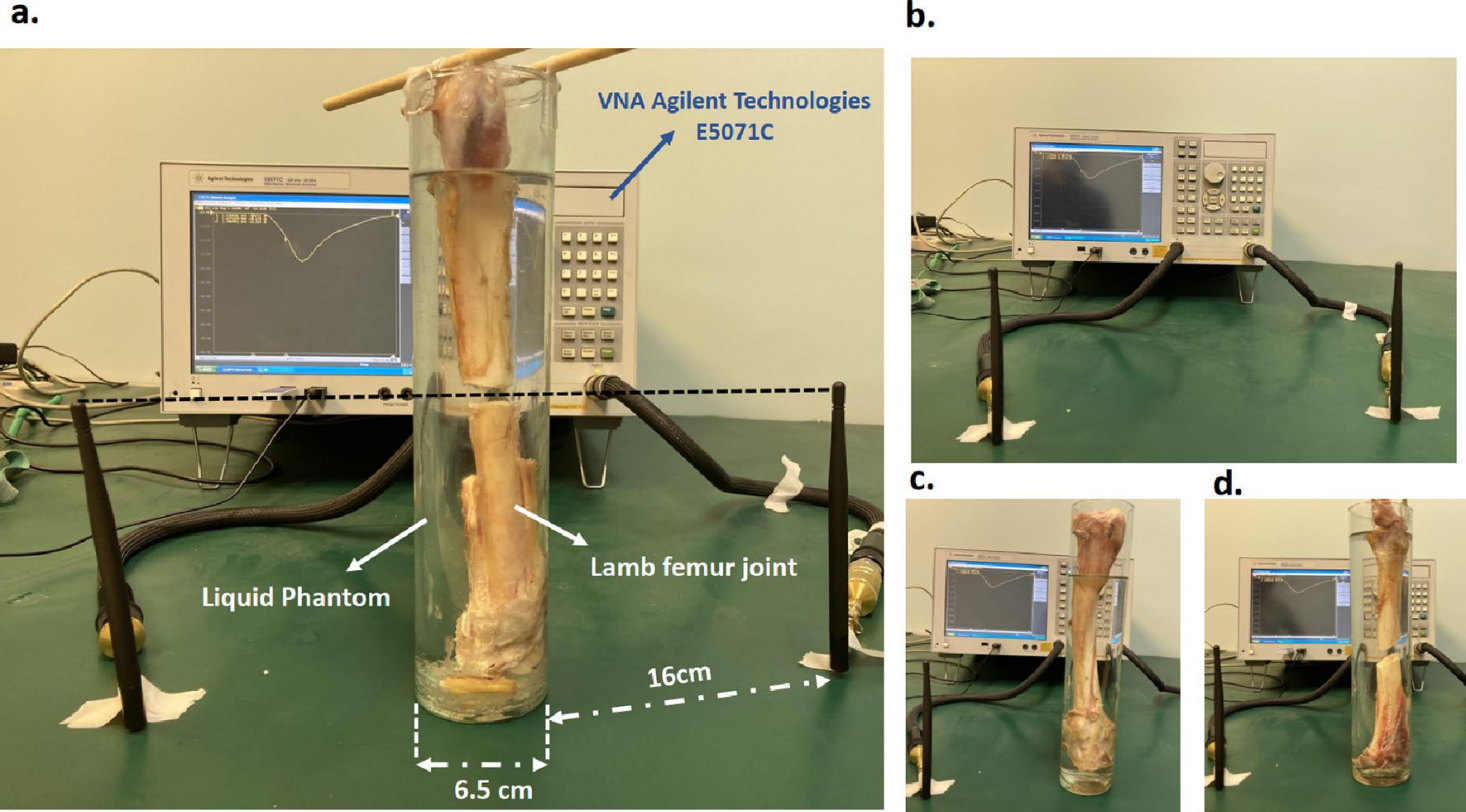
(**a**) Ex-vivo measurement on a sheep femur bone for Transverse fracture. (**b**) Free Space measurement setup using two commercially available monopole antennas connected to the VNA. The measurement was taken ex-vivo on a sheep femur bone with a (**c**) Normal, and (**d**) Oblique fracture.

### Ex-vivo measurement

In order to experimentally validate our results, we used sheep femur bones with a diameter similar to that of a human arm bone. These bones were sourced from a meat store. A commercially available phantom is used to mimic the human tissue at the ISM frequency band accurately. This phantom emulates the combination of electrical properties of skin, fat, and muscle at the ISM band through its permittivity and conductivity, the details of this phenomenon can be found in section 3.2. Noted that, in the muscle modeling, the effect of the blood is taken into account. We have prepared three different femur joints with nearly the same diameters for three types of measurements (transverse, oblique, and normal). Due to experimental difficulty, we did not conduct the measurements for the comminuted type of fractures. Refrigeration and hydration of the bones were maintained until the time of measurements. The distance between the monopoles to the bones was kept equal in all experiments (16 cm) as in the numerical simulation (see Fig. 6a). This distance ensures that the bones are placed at the far field of the antennas. It should be noted that the nearfield region of the antennas is avoided due to strong spatial variations of the electromagnetic field and undesired electromagnetic interference in this area, leading to lower detection accuracy. The diameter of the glass and bone was 6.5 cm and 20 mm, which was nearly equal to the human arm diameter.

**Figure 7:**
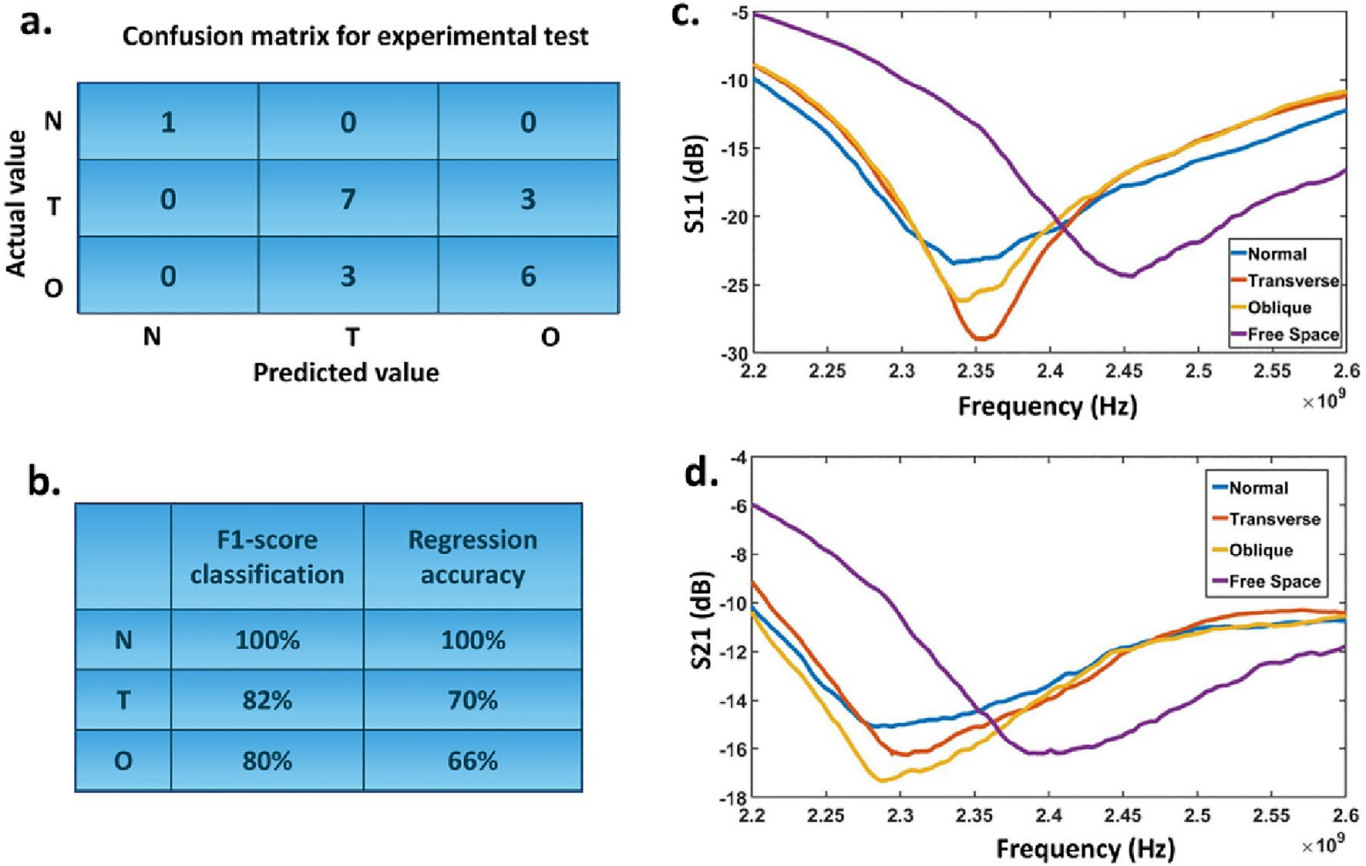
(**a**) Confusion matrix for the measurement results for three sets of experiments. N, T, and O correspond to normal bone, transverse and oblique fractures. (**b**) the amount of the F1-score for the classification of measurement results as well as the accuracy of regression. The amount of allowed deviation from the actual value is equal to $\pm 2$mm. The measurement reflection parameters (**c**) ${\mathrm{S_{11}}}$ and (**d**) ${\mathrm{S_{21}}}$ corresponds to four different tests.

For the first measurement, we placed two monopole antennas in free space to measure the reflection parameters. Each of the antennas was connected to E5071C Agilent Technologies VNA, which was calibrated from 2-3 GHz (The ${\mathrm{S_{11}}}$ of the antennas had a resonance at 2.45) as depicted in Fig. 6b. Then, the normal bone (without fracture) is inserted into the phantom and placed between the antennas, which is depicted in Fig. 6c. The S-parameters were taken from the VNA and entered as input for the DNN. Our designed network correctly classified it as a normal bone (see Fig. 7a).

For the second set of measurements, a transversal cut is made in the mid-section of the bone (see Fig. 6a). We induce cracks of varying lengths by adjusting the upper part of the bone with our designed fixture and fixing the lower part into the glass. By changing the crack length, the amount of the shift in the resonance frequency will be changed; thus, our network featuring two outputs, classification and regression (as shown in Fig. 3), can correctly distinguish the fracture type and simultaneously estimate the crack’s length. In the confusion matrix presented in Fig. 7a, the number of correct classifications is shown in the main diagonal of the matrix. There are 7 correct classifications out of 10 for transverse fractures. The wrong detection is dedicated to the smaller crack length. Figure 7b shows the amount of F1-score for all the experimental cases. The amount of the defined accuracy for the regression (fracture length) is also summarized in Fig. 7b. To derive these accuracy scores, we considered the amount of acceptable deviation from the actual value equal to $\pm 2$mm. Noticeably, our measured values of fracture lengths exhibit a mean regression error of 1.84 mm and a standard deviation of 1.26 mm. Also, by decreasing the length of the crack, the amount of the loss will be increased. Since we use non-invasive electromagnetic radiations to characterize the dielectric properties of the bone, very small changes in the crack length cannot make a visible shift to the resonance frequency. Therefore, our DNN is not capable of correctly estimating small changes in the crack length.

For the final sets of measurements, an oblique cut was made in the mid-section of the bone (See Fig. 6d). Same as in the previous case, cracks of varying lengths are generated by altering the upper section of the bone. A total of 9 measurements were conducted by changing the fracture dimensions. According to the results presented in Fig. 7a, our DNN is correctly classified. On the other hand, we have the previous problem with the regression. The amount of error will be increased by decreasing the crack’s length. In the discussion section, we have suggested some ideas to overcome these shortcomings. Also, the measurements of the S-parameters are presented in Fig. 7c and d for four different types of experiments. Consequently, the above experimental results verify that the proposed “fully-automated” DNN approach can be considered a promising candidate for fast and non-invasive fracture diagnosis. Since we have generated large sets of data by changing the diameters of the human tissue, our designed DNN is expected to diagnose different types of fractures in various anatomical regions. The developed system will guide medical personnel in terms of diagnosis of fractures since it doesn’t need experienced staff.

## Discussion

In this paper, we have provided a simple, portable, non-invasive, and fully-automated bone fracture diagnosis system for the first time. The output of the designed DNN can simultaneously classify the fracture types and estimate the length of the crack. The classification results are encouraging, while the regression results show comparable errors in some cases. Table 1 shows a comparison between this work and other studies on bone fracture diagnosis using microwaves. It reveals that, while the previous studies focused on microwave imaging of fractured bones, we developed a method to detect and classify bone fractures (without providing images) from microwave measurements. This would lead to faster diagnosis because there is no need to scan the bones under test to produce an image. However, imaging provides more comprehensive information about the body under test.

In the following, we will discuss some challenges and suggestions to address them.

1. According to the experimental results, small changes in the crack’s length cannot be distinguished by the network. Since we have used the ISM frequency band for simplicity and commercially available antennas, this frequency range may not be sufficient to distinguish small crack changes. Our results show that for the crack dimensions between 2 to 6 mm, the DNN regression output generates the same results. Therefore, to have a higher potential resolution to resolve smaller fractures, the transition toward a higher microwave frequency is needed. SANTOS et al.^13^ proposed a microwave imaging technique for the diagnosis of fractures in superficial bones. The experimental results demonstrate that at the 8.3–11.1 GHz frequency range, transverse fractures as small as 1 mm can be detected.
2. There is a trade-off between the penetration depth in biological tissues and decreasing the wavelength. Despite having a better regression resolution by increasing the frequency, the EM wave can not penetrate to the innermost layer, such as bones. Therefore, our simulation models will no longer correctly emulate the actual human tissue modeling. In order to cope with the low regression resolution, we suggest a frequency band between 10–20 GHz.
3. The next challenge is the VNA calibration. Before starting any experiment, the VNA should be calibrated to avoid the RF cable bending effects. Otherwise, the results may not be reliable since the RF cable bending can generate undesirable phase and amplitude fluctuations in the S-parameters profile. Therefore, the DNN may incorrectly classify the fracture type.
4. We did not consider the location of the fracture as a variable in the data preparation step to avoid network complexity. Therefore, we fixed the location of the fractures aligned with the location of the antenna source both in simulations and experiments. The black dashed line in Fig. 6a shows this alignment. Due to our simulations, large misalignments can cause fluctuations in the reflection parameters. Accordingly, it is recommended to keep the top of the monopoles aligned with the location of the fractures (this is possible when the patients and doctors can approximately estimate the fracture location.) Alternatively, the deep learning algorithm would need to learn the location of fractures based on the input data, which would significantly increase the complexity of the network.
5. Since it was impossible to use human bones for the experiments, we used animal bones. We observed that the actual permittivity and conductivity of the sheep’s femur might differ from those used in our DNN. In the simulations, we have inserted the permittivity and conductivity of the human bone, which may lead to some errors in the experiments regarding the regression. Furthermore, the animal joints used for the study were not exactly cylinders, and some variations in the S-parameters are expected due to imperfections and non-uniformities of the bones.
6. It should be accepted that, in reality, the human tissue properties are different from patient to patient. Factors such as age, gender, and the amount of water will cause some small variations in the permittivity and conductivity of different parts of the body^45,46^. Due to these variables, a wider range of data must be considered, resulting in an increase in the complexity of the inverse network.
7. In our scenario, the shift of the resonance frequency in the S-parameters was negligible, and the tissue properties were consistent over such small deviations. Therefore, we have used the fixed human tissue and bone permittivity and conductivity in the entire study. When the shift is wider, the simulation demands the assumption of a frequency-dependent dispersion model for human tissue properties. The multi-term Cole-Cole model^46^ and Gabriel’s measurement results for the frequency range of 1–4 GHz^47^ can be adopted.
8. There is a possibility of using the proposed system as a portable one. By using a simple WiFi module connected to the portable network analyzer, the S-parameters results can be transferred to the DNN input. The network can be established either on a Lab top or a smartphone. Then, the output of the network can be displayed on the screen of the device. This system can be installed inside any EMS vehicle without requiring an expert radiologist’s assistance for the diagnosis process.
9. Some works^30,48,49^ have investigated the sensor-type antenna design for medical applications by considering the acceptable amount of SAR^50,51^. In their work, the reflection characteristic of the antenna has a sharp resonance to assist easy distinction and interpretation in various medical applications. Based on the FCC guidelines, the SAR should be less than 1.6 W/Kg, which is defined as the amount of EM energy absorbed by body tissues. Since we used the commercial monopole antenna, it eliminates the need for any additional concerns regarding the amount of SAR.

**Table 1:** Comparison between recent studies on bone fracture diagnosis using microwaves.

Reference	Frequency	Probe/sensor	Need for scanning the bone	Output
^13^	8.3–11.1 GHz	Vivaldi antenna	Yes	Image of fractured bone
^14^	1–6.5 GHz	Horn and vivalid antennas	Yes	Image of fractured bone
^15^	2.45 GHz	Planar ring resonator	Yes	Image of fractured bone
This work	2.4 GHz	Monopole antennas	No	Detection and classification of fractures

## Conclusion

A DNN algorithm for simultaneous bone fracture classification and crack length estimation is implemented to develop a fully automated bone fracture diagnosis system using the ISM frequency band. This portable system is suitable for emergency situations to help with fast diagnosis when expert radiologists are not available. To circumvent the problem of labeling and data collection from hospitals, we used the S-parameters profile for DNN training instead of X-ray images. Based on numerical simulations, the loss function of the training and validation sets for both classification and regression verified that our DNN was successfully trained without overfitting. Also, the confusion matrix for the test dataset demonstrated that our DNN produces accurate and reliable fracture classification results.

To experimentally validate our results, sheep femur bones with a diameter similar to a human arm bone were used. A liquid phantom is utilized to mimic the exact human tissue model at the ISM frequency band. Our experiments demonstrated that the proposed network successfully classifies several fracture types. Also, it can successfully estimate the dimensions of the cracks for larger crack lengths. It should be emphasized that our DNN has the ability to diagnose different types of fractures in various anatomical regions.

We believe that our proposed fully-automated approach can pave the way for inexpensive, compact, and portable fracture diagnosis since it needs only two simple monopole antennas coupled through a VNA. In fact, it is conceivable that with proper training, our detection modality may be able to detect hairline fractures which are typically difficult to detect using X-rays.

## Data Availability

The data that supports the findings of this study are available from the corresponding author (V.N.) upon reasonable request.
